# Investigation of an Allosteric Deoxyhypusine Synthase Inhibitor in *P. falciparum*

**DOI:** 10.3390/molecules27082463

**Published:** 2022-04-11

**Authors:** Aiyada Aroonsri, Chayaphat Wongsombat, Philip Shaw, Siegrid Franke, Jude Przyborski, Annette Kaiser

**Affiliations:** 1 National Center for Genetic Engineering and Biotechnology (BIOTEC), National Science and Technology Development Agency (NSTDA), Khlong Luang 12120, Thailand; aiyada.aro@biotec.or.th (A.A.); chayaphat@biotec.or.th (C.W.); philip@biotec.or.th (P.S.); 2 Interdisziplinäres Forschungszentrum IFZ, Heinrich-Buff-Ring 26-32, 35392 Giessen, Germany; siegried.franke@ernaehrung.uni-giessen.de (S.F.); jude.przyborski@ernaehrung.uni-giessen.de (J.P.); 3 Medical Research Centre, University of Duisburg-Essen, Hufelandstrasse 55, 45147 Essen, Germany

**Keywords:** chemogenomic profiling, hypusine, bromobenzothiophene, deoxyhypusine synthase, *glmS* riboswitch, allosteric inhibitor

## Abstract

The treatment of a variety of protozoal infections, in particular those causing disabling human diseases, is still hampered by a lack of drugs or increasing resistance to registered drugs. However, in recent years, remarkable progress has been achieved to combat neglected tropical diseases by sequencing the parasites’ genomes or the validation of new targets in the parasites by novel genetic manipulation techniques, leading to loss of function. The novel amino acid hypusine is a posttranslational modification (PTM) that occurs in eukaryotic initiation factor 5A (EIF5A) at a specific lysine residue. This modification occurs by two steps catalyzed by deoxyhypusine synthase (*dhs*) and deoxyhypusine hydroxylase (DOHH) enzymes. *dhs* from *Plasmodium* has been validated as a druggable target by small molecules and reverse genetics. Recently, the synthesis of a series of human *dhs* inhibitors led to 6-bromo-*N*-(1*H*-indol-4yl)-1-benzothiophene-2-carboxamide, a potent allosteric inhibitor with an IC_50_ value of 0.062 µM. We investigated this allosteric *dhs* inhibitor in *Plasmodium*. In vitro *P. falciparum* growth assays showed weak inhibition activity, with IC_50_ values of 46.1 µM for the Dd2 strain and 51.5 µM for the 3D7 strain, respectively. The antimalarial activity could not be attributed to the targeting of the *Pfdhs* gene, as shown by chemogenomic profiling with transgenically modified *P. falciparum* lines. Moreover, in dose-dependent enzymatic assays with purified recombinant *P. falciparum* *dhs* protein, only 45% inhibition was observed at an inhibitor dose of 0.4 µM. These data are in agreement with a homology-modeled *Pfdhs*, suggesting significant structural differences in the allosteric site between the human and parasite enzymes. Virtual screening of the allosteric database identified candidate ligand binding to novel binding pockets identified in *P. falciparum* *dhs*, which might foster the development of parasite-specific inhibitors.

## 1. Introduction

Almost 50 years after the establishment of the WHO’s Global Malaria Eradication Programme, significant decreases in mortality rates have been achieved and an increasing number of countries have been certified as malaria-free. This success prompted a bold plan to reduce 90% of deaths caused by malaria until 2030 [1] and to eliminate malaria in 35 more countries. These goals can only be achieved by a continuous strategy attacking the parasite with novel drugs at different stages of its life cycle due to developing resistance against registered drugs. Although the first vaccine, RTS,S/AS01_E_ [2], against malaria has been recommended by the WHO, open questions about its modest efficacy and its complex regimen remain [3]. However, novel strategies such as chemoprotection in combination with sporozoite vaccination during controlled human malaria infection (CMI) show promising results [4]. Nevertheless, small-molecule drugs will be necessary to eradicate malaria.

The discovery of novel targets in *Plasmodium* has been accelerated by molecular genetics, i.e., genome editing by CRISPR-Cas9 [5] and reverse genetic tools that combine *trans*- or *cis*-acting genetic methods, which attenuate the expression of a gene of interest. Trans-acting reverse genetic tools are short hairpin RNA (shRNA) [6] or antisense oligonucleotides that are engaged with the mRNA and cause a posttranscriptional attenuation of gene expression. However, both tools are not yet sufficiently mature in *Plasmodium* due to the absence of a canonical RNAi regulated pathway in the parasite [6] and off-targeting effects in the latter. In contrast, classical *cis*-acting reverse genetic tools do not show off-targeting effects; however, manipulation of the parasite’s genome is required that involves transfection of a plasmid encoding the sequence of a gene of interest mediating homologous recombination [6] and a selectable marker. A broad spectrum of *cis*-acting reverse genetic tools has been developed which enable conditional attenuation of expression of a gene of interest [7] or inducible knockouts with the dimerizable Cre (DiCre) system [8].

**Figure 1:**
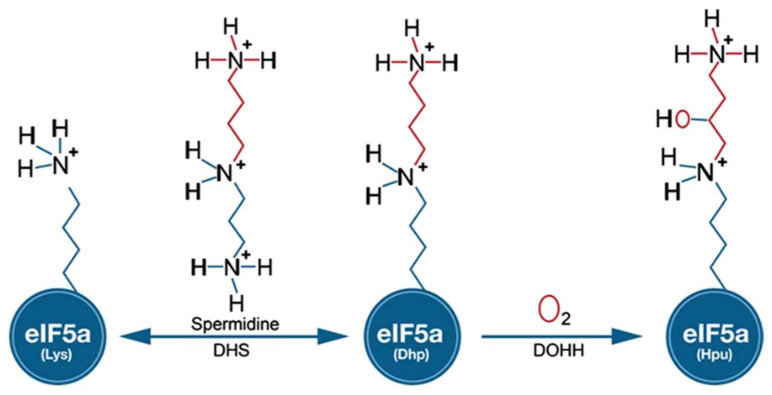
Biochemical pathway leading to the formation of hypusine in EIF5A protein: In the first step of the pathway, DHS transfers an aminobutyl moiety from the substrate spermidine to a specific lysine residue in the EIF5A precursor protein to form deoxyhypusine. In the second step, deox-yhypusine hydroxylase (DOHH) completes hypusine biosynthesis, introducing a hydroxyl group into the side chain.

One of the most widely used *cis*-acting tools for regulating gene expression at the RNA level in *Plasmodium* is the *glmS* riboswitch. Originally, this regulatory element was discovered in the 5′-UTR of glucosamine-6-phosphate synthase in bacteria [9]. In principle, modulation of gene expression is induced in response to glucosamine-6-phosphate (GlcN6P) that activates the autocatalytic cleavage of RNA through ribozyme resulting in mRNAs with a short half-life in vivo. Gene expression can be monitored by inserting the *glmS* riboswitch downstream of the gene of interest (including drug targets) by an integration vector [10] that is tagged. Among the potential new drug targets, we identified canonical genes involved in the biosynthesis of the unique amino acid hypusine that occurs at lysine 50 in eukaryotic initiation factor 5A (eIF5A) in different human malaria parasites. The modification to hypusine requires two sequential steps: (i) NAD-dependent transfer of the aminobutanal moiety from the triamine spermidine to a specific lysine residue in the eIF5A precursor protein by deoxhypusine synthase (DHS) and (ii) introduction of a hydroxyl group into the side chain by deoxyhypusine hydroxylase (DOOH) [11] (Figure 1).

Different small-molecule inhibitors targeting either *dhs* or DOHH arrest the growth of the malaria parasite [12]. To further investigate the essential function of the eIF5A precursor protein and the activating *dhs* enzyme in the pathogenic blood cell stage, targeted gene disruption was performed in the rodent malaria parasite *P. berghei* [13]. However, only transgenic parasites with either *eIF5A* or *dhs* in their episome were obtained, suggesting a vital function in murine malaria blood stages. These results motivated the establishment of a stable *glmS* transgenic line with an integrated, recombinant GFP tagged *dhs* gene [14]. These transgenic parasites harboring the *glmS* riboswitch downstream of the *Pfdhs* gene showed reduced hypusine biosynthesis and a growth defect [14], confirming that the *dhs* gene is essential. Moreover, these *glmS* transgenic lines enable chemogenomic profiling for assessing the on-target specificity of *dhs* inhibitors.

**Figure 2:**
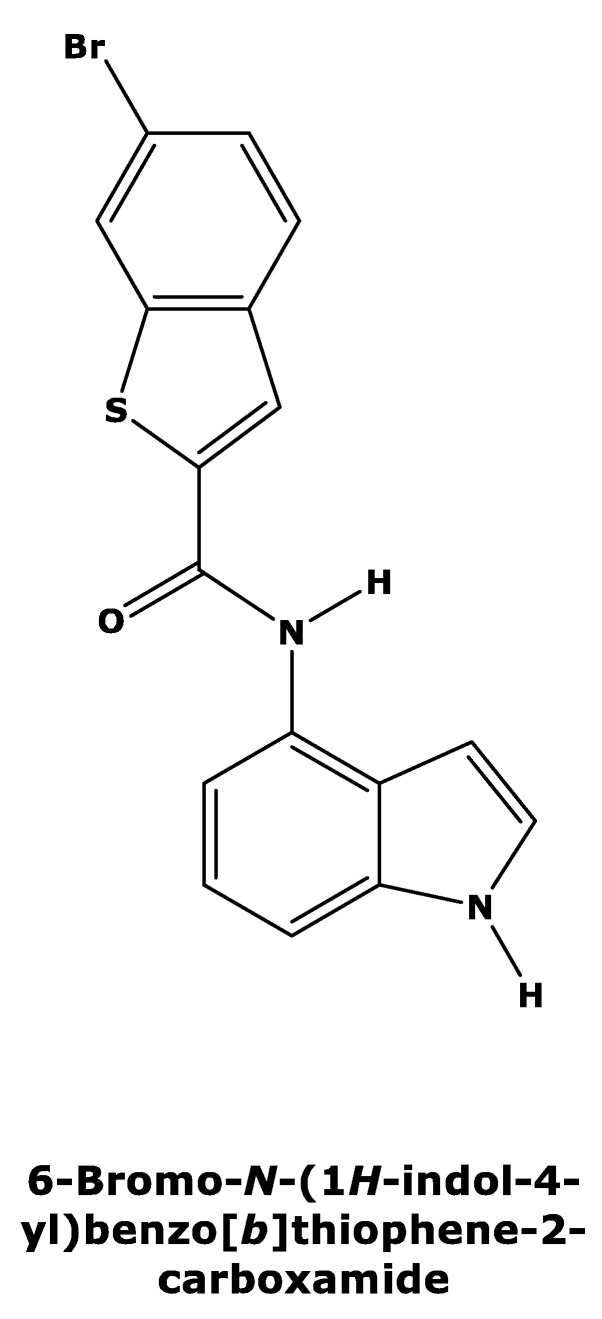
Two-dimensional structure of compound 6-bromo-*N*-(1*H*-indol-4yl)-1-benzothiophene-2-carboxamide (PubChemCID146014943).

Recently, Tanaka et al. [15] published a new series of potent allosteric inhibitors of human deoxyhypusine synthase to prevent the activation of EIF5A and subsequent translation of mRNAs in proliferating tumor cells. The most potent inhibitor was 6-bromo-*N*-(1*H*-indol-4yl)-1-benzothiophene-2-carboxamide (Figure 2) [15], which acts on a novel allosteric site distinct from spermidine mimetics inducing a conformational change.

Here, we investigated the inhibition of *Plasmodium dhs* by this allosteric *dhs* inhib-itor. In vitro growth of chloroquine-sensitive and chloroquine-resistant strains were weakly inhibited, suggesting a lack of specific target engagement. Chemogenomic profiling confirmed the lack of *Plasmodium dhs* target specificity. Dose-dependent inhibitory assays with the purified *Plasmodium dhs* enzyme demonstrated 45% inhibition at an inhibitor dose of 0.04 µM, which is substantially weaker compared with human *dhs*. The difference in inhibitor potency can be explained by structural differences in the allosteric site between the human and parasite *dhs* enzymes, as revealed by in silico analyses. These differences can be exploited for the design of parasite-specific allosteric inhibitors.

## 2. Results

### 2.1. 6-Bromo-N-(1H-indol-4yl)-1-benzothiophene-2-carboxamide Has No Impact on Parasite Growth

Since one of the main biological functions of *dhs* is to promote cell proliferation [16], inhibition of growth was investigated in vitro in two *P. faciparum* strains with a different sensitivity to chloroquine. To determine the IC_50_ values, an SYBR Green 1(SG)-based in vitro method was employed. The determined IC_50_ value for 6-bromo-*N*-(1*H*-indol-4yl)-1-benzothiophene-2-carboxamide is 46.1 µM for the chloroquine-resistant Dd2 strain, which confers the chloroquine-resistant isoform of the chloroquine resistance transporter (CRT), (Table 1). In contrast, the IC_50_ values of the antimalarials amodiaquine and chloroquine are 0.031 µM and 0.66 µM, respectively (Table 1). These data indicate that the compound does not inhibit in vitro growth of the chloroquine-resistant Dd2 *falciparum* strain with a level of potency expected for the inhibition of an essential gene target.

The growth of the chloroquine-sensitive 3D7 strain was inhibited by the DHS allosteric inhibitor to a similar degree with an IC_50_ value of 51.5 µM (Table 1). In contrast, the antimalarials amodiaquine and chloroquine had IC_50_ values of 0.066 µM and 0.03 µM, respectively. In sum, the weak growth inhibition for both strains indicates that the DHS allosteric inhibitor has no significant target engagement in both *P. falciparum* strains.

### 2.2. 6-Bromo-N-(1H-indol-4yl)-1-benzothiophene-2-carboxamide Does Not Primarily Target the DHS Protein in P. falciparum

**Figure 3:**
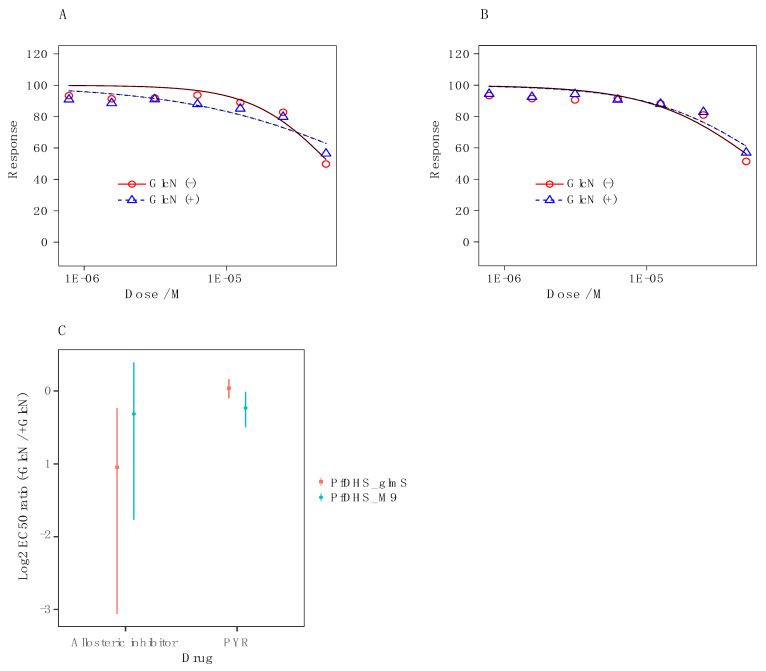
Dose–response growth inhibition curves with the allosteric inhibitor 6-bromo-*N*-(1*H*-indol-4yl)-1-benzothiophene-2-carboxamide against: (**A**) Transgenic *Pfdhs*_*glmS* parasites and (**B**) ribozyme-inactive transgenic *Pf*DHS_M9 parasites with cotreatment of 2.5 mM glucosamine (GlcN) (blue triangles) or without co-treatment (red circles). (**C**) The estimated log_2_ ratio of EC_50_ between the +GlcN (blue points) and −GlcN (red points) conditions together with CI_95_ is shown for the allosteric inhibitor and the pyrimethamine control in transgenic parasites, i.e., *Pfdhs*_*glmS* and the inactive mutant. *p*-Values comparing log_2_ EC_50_ (−GlcN/+GlcN) estimate: 0.58 *Pfdhs*_*glmS* (pyrimethamine): 1 × 10^−4^ *Pfdhs*_M9 (pyrimethamine); 0.81 *Pfdhs*_*glmS* (allosteric inhibitor); 4 × 10^−8^ *Pfdhs*_M9 (allosteric inhibitor).

It is possible that the weak growth inhibition of *P. falciparum* parasites by 6-bromo-*N*-(1*H*-indol-4yl)-1-benzothiophene-2-carboxamide can still be attributed to *dhs* targeting, since the spermidine analog N1-guanyl-1,7-diaminoheptane demonstrates modest potent antimalarial activity via inhibition of *P. falciparum dhs* [12,14]. Therefore, we tested *dhs* targeting by chemogenomic profiling with the transgenic *P. falciparum* parasites *Pf*DHS_*glmS* and the ribozyme-inactive *Pfdhs*_M9. In chemogenomic profiling, GlcN co-treatment sensitizes the *Pfdhs*_*glmS*, but not the Pf*dhs*-M9 parasite to *dhs*-targeting compounds [14]. The data for assays with 6-bromo-*N*-(1*H*-indol-4yl)-1-benzothiophene-2-carboxamide show that co-treatment with 2.5 mM GlcN does not sensitize the *Pfdhs*_*glmS* (Figure 3A) or *Pfdhs*-M9 parasite (Figure 3B). Furthermore, the log_2_ EC_50_ (−GlcN/+GlcN) ratio is not significantly greater than 0 for transgenic parasites treated with the DHS allosteric inhibitor or the control antimalarial compound pyrimethamine, an inhibitor of dihydrofolate synthase, an enzyme that participates in folic acid biosynthesis (Figure 3C). We conclude from these data that 6-bromo-*N*-(1*H*-indol-4yl)-1-benzothiophene-2-carboxamide does not inhibit *P. falciparum* growth via targeting of *dhs*.

### 2.3. Inhibitor Studies with Recombinant, Purified dhs from P. falciparum Suggest a Different Allosteric Site in the Enzyme of the Parasite

**Figure 4:**
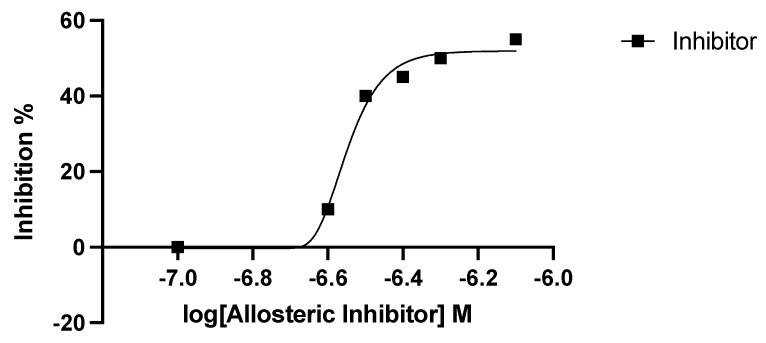
Dose–response curve of the allosteric inhibitor showing the log doses (x axis) versus % inhibition (y axis) in non-linear regression.

Next, dose-dependent enzyme inhibition with different concentrations in the range between 20 µg and 100 µg of 6-bromo-*N*-(1*H*-indol-4yl)-1-benzothiophene-2-carboxamide was performed. In these experiments, we employed purified enzyme preparations of *P. falciparum* DHS and analyzed the peptide hydrolysates for the presence of deoxyhypusine by GC/MS. First, relative quantification was employed to determine the inhibitory activity of the allosteric inhibitor. Thus, deoxyhypusine was quantified with external calibration as described in [17]. Thereby, a relative *dhs* inhibition of approximately 45% at an inhibitor concentration of 0.4 µM relative to the positive control without an inhibitor could be detected (Figure 4). Table 2 summarizes the determined IC_50_ values of *P. falciparum dhs* and the human orthologue. The IC_50_ value of *dhs* from *P. falciparum* was approximately five times higher compared with that of the human enzyme, with only 45% maximum inhibition. These data suggested significant differences in the allosteric site between the human and parasite *dhs* proteins.

**Figure 5:**
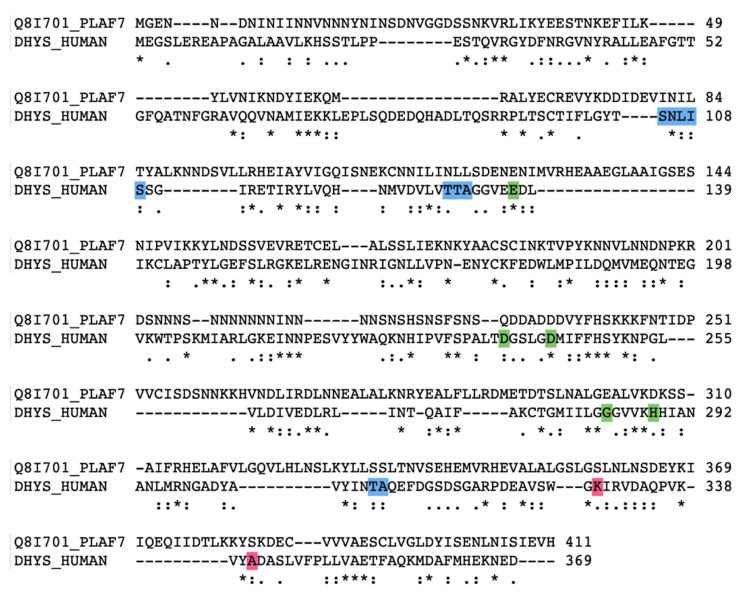
Amino acid alignment of *P. falciparum* DHS (first lane) and the human orthologue (second lane) performed with the Clustal Omega alignment tool. Gaps (-) were introduced to obtain maximum alignment. Asterisks label amino acid identities, colons (:) and dots (.) label amino acid similarities. Amino Acids marked in blue represent nucleotide binding sites in human DHS. Possible ligand binding sites are colored in green. Lys328 and Ala351 are part of the active site and shown in red color.

The aforementioned results challenged us to identify a putative allosteric site in the DHS enzyme of the parasite. Indeed, we localized four possible binding sites in the amino acid alignment that were present in human DHS (marked in green) (Figure 5). These binding sites, however, do not occur in the parasite enzyme (Figure 5) with one exception, i.e., Asp238. Tanaka et al. [15] showed in a three-dimensional model (3D) that was obtained from the co-crystallized DHS structure with the inhibitor 6-bromo-*N*-(1*H*-indol-4yl)-1-benzothiophene-2-carboxamide that five amino acids of the allosteric binding site in human DHS are involved in the inhibitor interaction: (i) Asn 267 and Lys329 form hydrogen bonds with the inhibitor; (ii) Asp238 interacts via a crystal water molecule; (iii) His288 is of importance in the NH-π interaction; and (iv) Leu281 associates with the bromide of the thiophene ring. However, of the five interacting amino acids, only Asp238, Asp267 and Leu281 are present in parasite DHS.

### 2.4. In Silico Prediction of Allosteric Sites in P. falciparum DHS

**Figure 6:**
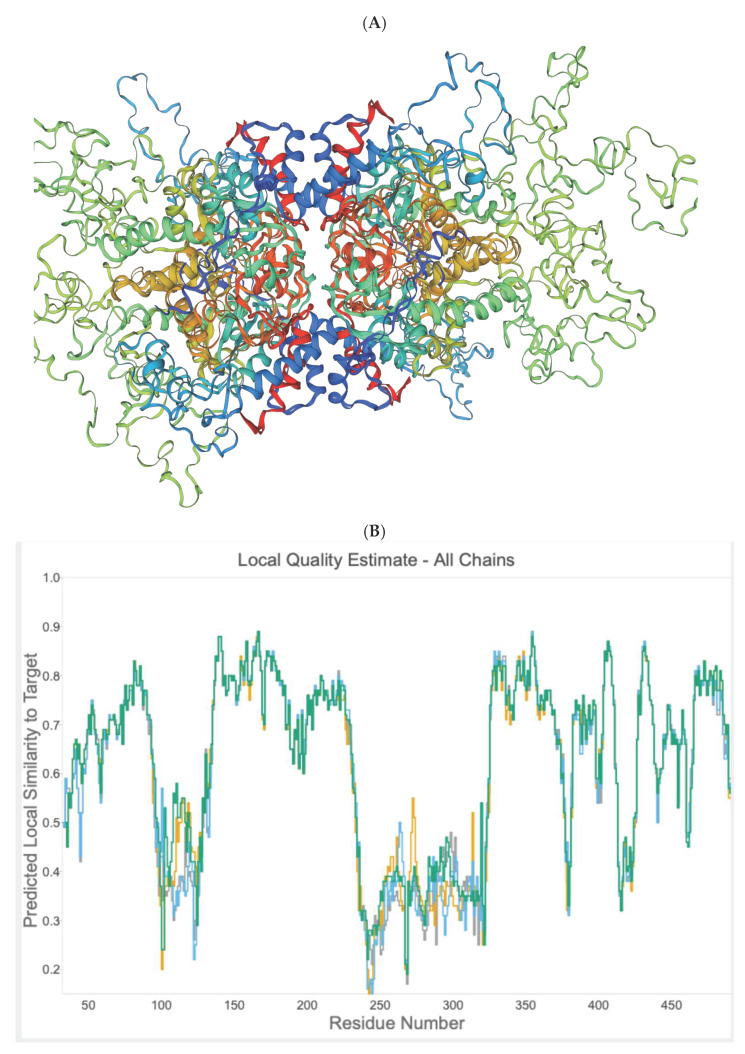

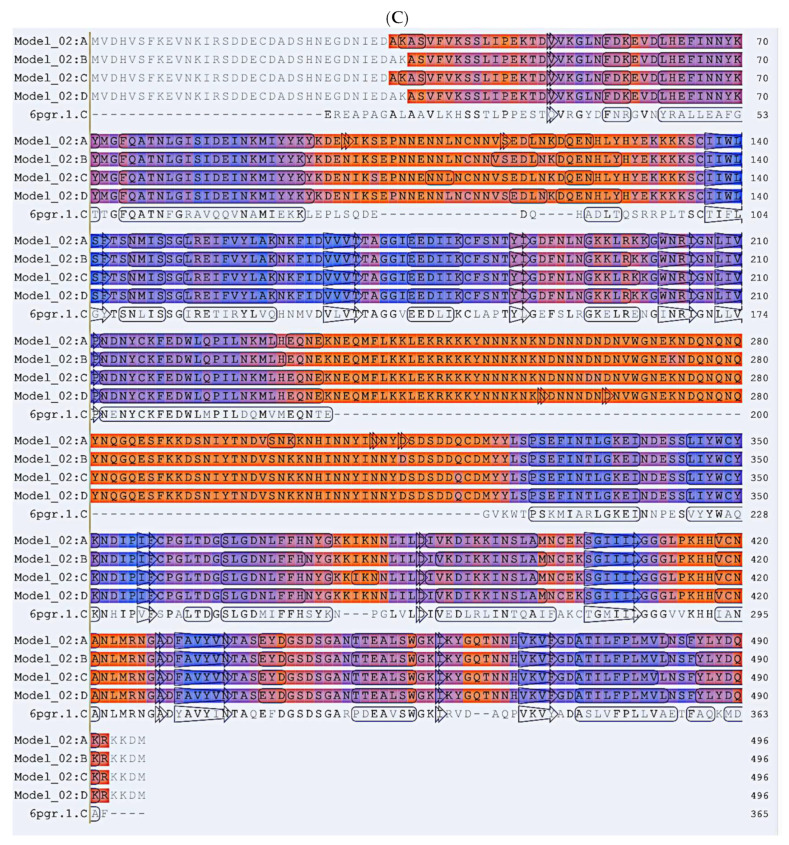
(**A**) In silico homology model of *Pf*DHS. Modelling was performed on the template alignment of human DHS co-crystallized with 6-bromo-*N*-(1*H*-indol-4yl)-1-benzothiophene-2-carboxamide (PDB 6pgr) and *Pf*DHS (PDB 1rlz.1A) generated by Swiss Model. The protein consists of four identical subunits shown in rainbow, i.e., red (subunit A), blue (subunit B), green (subunit C) and brown (subunit D). (**B**) Graph of local quality estimate for the homology model structure of *Pf*DHS. Q-MEAN score (y-axis) is plotted against *Pf*DHS residue number. Subunits of a putative tetramer are plotted on the same axes (Chain A: grey; Chain B: yellow; Chain C: cyan; Chain D: green). (**C**) Alignment of *P. falciparum* DHS strain D7 (PDB:1rlz.1A) and human DHS co-crystallized with 6-bromo-*N*-(1*H*-indol-4yl)-1-benzothiophene-2-carboxamide (PDB: 6pgr). Secondary structure elements are indicated on the protein residues (oblong, alpha helix; arrow, beta-sheet).

**Figure 7:**
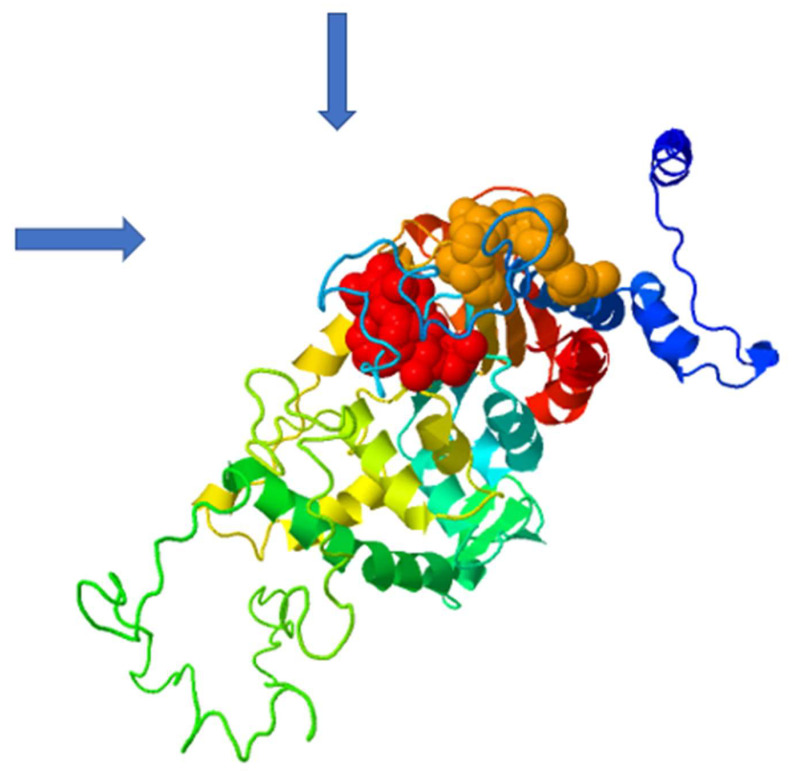
Structural prediction of two allosteric binding pockets (marked by blue arrows) predicted by the PASServer employing the 3D model of human DHS co=crystallized with the allosteric inhibitor. Binding Pocket 1 (red color) has the highest probability of 59.36% and a druggability score of 0.007. Pocket 2 (brownish color) has a probability score of 58.2% and a druggability score of 0.228.

Based on the amino acid sequence of DHS from *P. falciparum* strain 3D7, the co-crys-tallized human DHS with the allosteric inhibitor was used as a template for 3D-structure modeling [15,18] using the Swissmodel Expasy Database [19] and the AlphaFold Protein Structure Database [20]. The resulting homology 3D model is depicted in Figure 6A and is based on the alignment with the cocrystallized human DHS and the allosteric inhibitor. The QMEAN score of 0.62 suggests the model’s reliability (Figure 6B). All four chains demonstrate a QMEAN score above 0.62 except in the region of the insertion loops. The resulting homology 3D structure (Figure 6A) proves the homotetrameric structure of the plasmodial protein. Next, a screen for predicted allosteric sites employing the Protein Allosteric Site Server (PASServer) [21] was performed. Three different allosteric pockets were identified with a probability of 59.36%, 58.52% and 47.11%. The pocket with the highest probability, i.e., 59.36% comprises the following amino acids: Ile137, Glu130, Asp 165, Val166, Phe357, Lys162, Val114, Glu123, Tyr127, Lys133, Lys132, Asn108, Lys 134, Leu397, Lys393, Cys401, Asp121, Asn 400, Asn352, Lys131, Ser396, Asp353, His125, Tyr129, and Pro355. The 3D structure is depicted in Figure 7 with the predicted pocket 1 that is marked in red. However, the druggability score for this pocket is only 0.007. In contrast, pocket 2 (brownish color) with the lower probability of 58.52%, has a much higher druggability score of 0.228. Pocket 2 covers the following amino acids: Tyr9, Lys93, Glu97, Asp96, Tyr 488, Glu402, Lys134,Tyr92, Ser101, Lys491, Ser135, Lys133, Tyr94, Lys95, Asp98, and Lys403.

**Figure 8:**
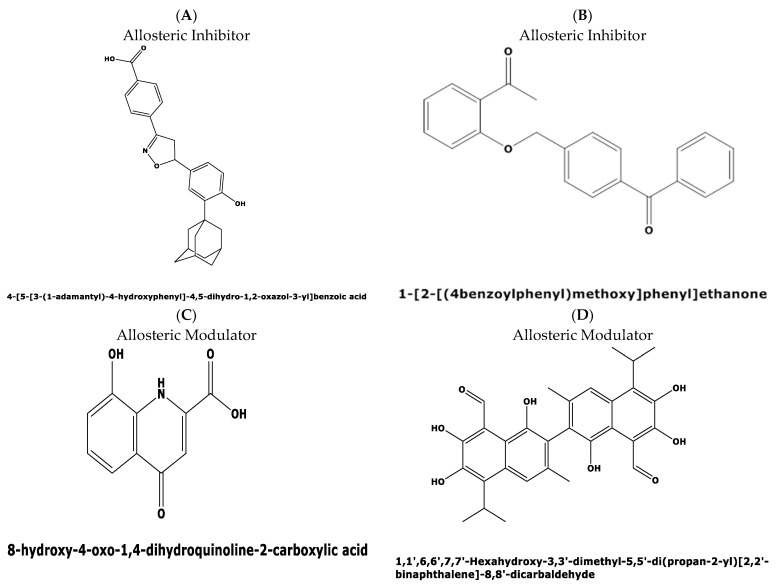
Predicted allosteric modulators and similar allosteric metabolites for binding pockets 1 and 2 in the plasmodial DHS protein obtained from the Allosteric Database and Allobase. (**A**) 4-[5-[3-(1-adamantyl)-4-hydroxyphenyl]-4,5-dihydro-1,2-oxazol-3-yl]benzoic acid with the top Alloscore of 8.61. (**B**) 1-[2-[(4benzoylphenyl)methoxy]phenyl)]ethanone is an allosteric inhibitor with an Alloscore of 8.02. (**C**) Xanthurenic acid (4,8-Dihydroxyquinoline-2-carboxylic acid represents a known, endogenous metabolite to the allosteric inhibitor A. (**D**) Data mining in the Allobase shows significant similarity of compound B to Gossypol (1,1′,6,6′,7,7′,-Hexahydroxy-3,3′-dimethyl-5,5′-(dipropan-2-yl)[2,2′-binaphtalene]-8,8′-dicarbaldehyde) as an allosteric modulator.

The divergence in the druggability score between the top two candidate binding pockets encouraged us to perform a virtual screening for possible ligands binding to both pockets in the Allosteric Database [22] employing the CHEMBL Diversity library. This screening resulted in the identification of three allosteric modulators with the highest score of 8.61, and 8.02 for two other compounds, respectively. The three allosteric modulators obtained with top scores were: (i) 4-[5-[3-(1-adamantyl)-4-hydroxyphenyl]-4,5-dihydro-1,2-oxazol-3-yl]benzoicacid(MF: C_26_H_27_NO_4_; MW: 417.5 g/mol. Compound ID: CHEMBL: 476316) (Figure 8A); (ii) N-(3,4-dimethyl-1,2-oxazol-5-yl)-2-[2-[(3,3-dimethyl-2-oxopyrrolidin-1-yl)methyl]-4-[(4oxo-2-propyl-5,6,7,8.-tetrahy-dro-cyclo-hepta-[d]imidazol-3-yl)methyl]phenyl]-benzene-sulfon-amide (MF: C23H18O5, MG: 374.4 g/mol, ID: CHEMBL 12073) (iii) 1-[2-[(4benzoylphenyl)methoxy]phenyl]ethanone (MF: C22H18O3, MW: 330.4 g/mol, ID: CHEMBL1477069) (Figure 8B).

Interestingly, the first compound, a benzoic acid derivative, possesses antiproliferative activity against human HL60 cells, while the second compound with a benzene–sulfon–amide structure targets the human cysteinylleukotriene receptor1 [23] with anti-inflammatory properties suppressing leukotriene formation. The third compound perturbs the GNAS complex stimulatory G_s_-protein alpha subunit [24], a key element of the classical signal transduction pathway linking receptor–ligand interactions with the activation of adenylyl cyclase and a variety of cellular responses.

Next, mapping of the three compounds in the Allosterome database was performed. The 2D structure of each predicted allosteric modulator was searched in AlloFinder, comprising 17,323 endogenous allosteric metabolites and in KEGG, using the MACCS (Molecular Access Database) Finger-print of Open label Fastsearch algorithm (BIOVIA, Dassault Systems, Hannover, Germany). Surprisingly, the predicted allosteric inhibitor, i.e., compound **1** (Figure 8A), revealed similarity to the allosteric metabolite xanthurenic acid (Figure 8C) which is an inducer of gametogenesis of *Plasmodium* in the mosquito [25]. Secondly, the data mining of the predicted allosteric inhibitor (Figure 8B) in the Allofinder revealed gossypol (Figure 8D) as an allosteric metabolite with significant similarity [26]. Gossypol is a di-sesquiterpene natural product in the form of a functionalized binaphthyl and is isolated from cotton plants. It inhibits plasmodial lactate dehydrogenase, which is essential for energy metabolism.

## 3. Discussion

In recent years, understanding the molecular regulation of allosteric modulation has attracted considerable attention in drug discovery [27,28]. This is due to the benefits and versatility of allosteric modulators providing selective inhibition of the protein target while minimizing toxicity and undesirable side effects. To date, only 19 allosteric drugs have been approved by the FDA compared to 3700 approved drugs binding to an orthosteric site [28,29]. Computational approaches such as predicting the 3D structure of proteins [19,20] and their ligand binding sites and putative binders in silico [22,28] have fostered the development of allosteric drugs.

The relevance of allosteric research for combating infectious parasitic diseases has been demonstrated in the case of the American trypanosomiasis (Chagas disease), where pockets with allosteric potential were identified and characterized in a cysteine protease (cruzain) in efforts to inhibit the causative agent *Trypanosoma cruzi* [30]. In this context, it is interesting to note that *T. brucei* DHS is allosterically regulated by a dead paralog [30], which is catalytically inactive but essential for in vitro growth and activity. This result challenged us to perform a 3D-modeling approach in the Swiss Expasy Database [19] using the sequence of the regulatory DHS subunit of *T. brucei* and the co-crystallized human DHS structure from *P. falciparum* with the allosteric inhibitor. However, only the A chain has a Qmean score of 0.57, suggesting significant structural differences between plasmodial DHS and the enzyme from *T. brucei*. Moreover, the amino acid identity is only 34%. This result furthermore confirms the phylogenetic distance of the DHS protein between Apicomplexan parasites and Trypanosomatides.

The recent identification of an allosteric inhibitor, i.e., 6-bromo-*N*-(1*H*-indol-4yl)-1-benzothiophene-2-carboxamide, against human DHS prompted us to investigate its efficacy against the orthologue from *Plasmodium*. The DHS protein catalyzes the regulatory step in hypusine biosynthesis, activating eukaryotic initiation factor 5A (EIF5A), which in turn promotes translation of mRNAs with polyproline motifs [31] exhibiting a variety of biological effects. The most important targets in alleviating parasitic diseases are proteins that are involved with or associated with cell proliferation. Thus, inhibition of DHS might be a novel therapeutic option. N-1-guanyl-1,7-diaminoheptane (GC-7) is a small-molecule competitive inhibitor of DHS [32], but it lacks sufficient selectivity to be used as a drug due to its unspecific binding to other targets because of its structural similarity to spermidine, which is thought to play several biological roles. In an attempt to screen for novel inhibitors with higher selectivity than the spermidine mimetics, 6-bromo-*N*-(1*H*-indol-4yl)-1-benzothiophene-2-carboxamide was identified with potent inhibitory activity through binding to the allosteric site in human DHS [15]. The data from this study show that this allosteric inhibitor does not inhibit *P. falciparum* DHS protein. This was confirmed by a relative DHS inhibition of approximately 45% at an inhibitor concentration of 0.4 µM relative to the positive control. A further increase in the allosteric inhibitor dose did not prove dose-dependent inhibition. In contrast, CNI-1493, a competitive human DHS inhibitor, showed dose-dependent inhibition [33] of the parasite enzyme.

**Figure 9:**
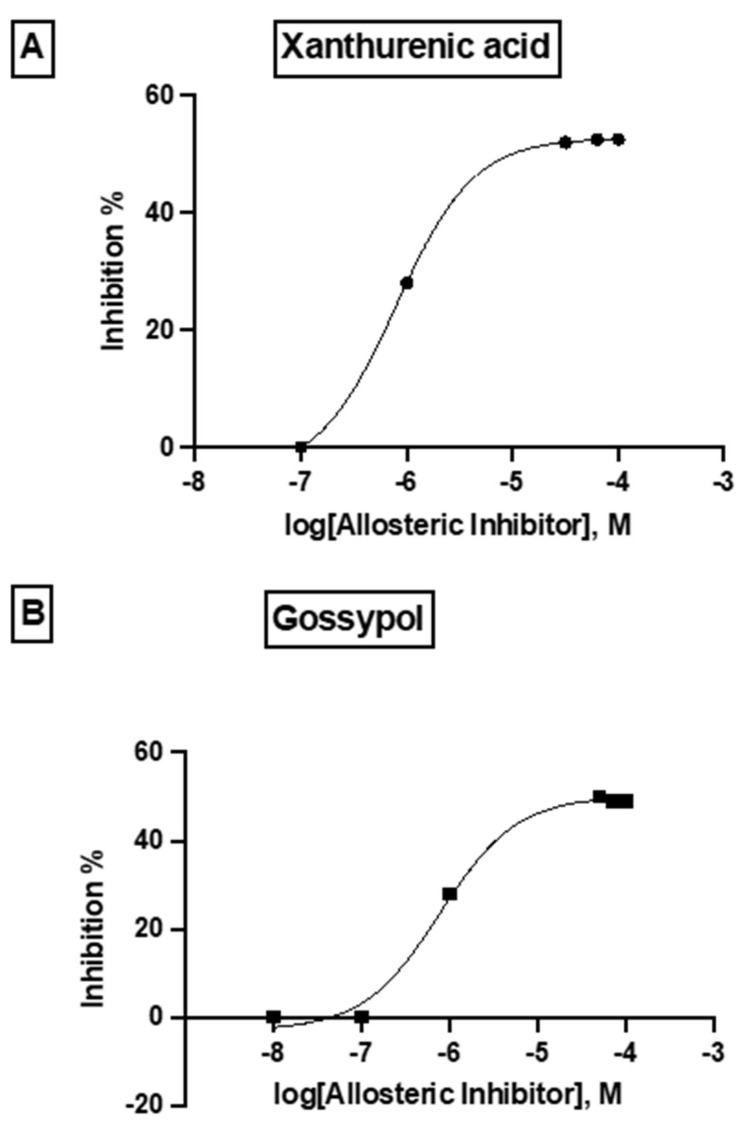
Dose–response curve of the allosteric metabolites xanthenuric acid and gossypol showing the log doses (*x* axis) versus % inhibition (*y* axis) in non linear regression.

The differences in the allosteric site between the human and parasite DHS proteins were apparent from the in silico 3D structure. Based on the in silico 3D structure, two allosteric binding pockets (Figure 7) were identified with a probability score of 59.36 and 58.52%, respectively. Interestingly, the pocket with a probability score of 58.52% has a higher druggability score of 0.228. However, this druggability score reflects a poorly druggable binding pocket (0.2–0.5) according to a histogram that was recently published by Farfán-López et al. [34]. The authors defined that druggable pockets are in the range between 0.5 to 0.7, while highly druggable pockets are in the range between 0.7 and 1.1 [34]. Therefore, we performed a “proof of principle” experiment for the two identified allosteric metabolites, i.e., xanthurenic acid and gossypol, since the top two candidate allosteric inhibitors were not commercially available. Indeed, both compounds did not show significant improved inhibition of the purified, recombinant DHS protein from P. falciparum. Xanthurenic acid showed 52% relative inhibition at a concentration of 0.35 µM, while 50% relative inhibition was obtained at a concentration of 0.42 µM gossypol. This resulted in an IC_50_ of 0.112 µM for xanthurenic acid and an IC_50_ of 0.310 for gossypol (Figure 9A,B). To improve the selectivity of these compounds, either the two allosteric inhibitors have to be synthesized for an assay or a molecular docking experiment has to be performed.

Novel allosteric binding sites were identified in *P. falciparum* DHS and several candidate interacting compounds were identified by virtual screening, suggesting that para-site-selective allosteric DHS inhibitors could be developed as novel antimalarial drugs.

## 4. Materials and Methods

### 4.1. Growth Inhibition of P. falciparum In Vitro Cultures and Determination of IC_50_ Values for Dd2 and 3D7 Strains Using a SYBR Green 1(SG)-Based In Vitro

IC_50_ values were determined according to a method established by Dery et al. [35] with modifications. In principle, the assay employs the incorporation of the dye SYBR Green 1 into parasite DNA. After freezing and thawing of parasite cultures, SYBR Green 1 fluorescence can be measured to determine IC_50_ values and thus test the susceptibility to antimalarial drugs. IC_50_ values of amodiaquine and chloroquine were determined in control experiments. Emission of fluorescence was measured with the multi-mode plate reader CLARIOSTAR Plus (BMG Labtech, Ortenberg, Germany).

### 4.2. Dose–Response Growth Inhibition Assays

For dose–response growth inhibition assays, transgenic *Plasmodium falciparum Pf*DHS_*glmS* and the inactive variant *Pf*DHS_M9 [14] were employed and provided by Aiyada Aroonsri. Dose–response assays were conducted as described previously [14,36]. Ring-stage synchronized parasites were diluted with complete medium and human erythrocytes to 1% parasitemia and 2% hematocrit. Ten microliters of the diluted com-pound were transferred into individual wells of 96-well microtiter plates. In addition to the tested compound, 10 µL of 1 × PBS or 25 mM GlcN dissolved in 1× PBS were added into each well. Samples of 80 µL of the ring-stage synchronized parasite, or uninfected erythrocyte suspension, were added and the plates subsequently incubated in a gassed chamber at 37 °C for 48 h. Cells were lysed with 100 µL of SYBR Green I lysis buffer and fluorescence was measured with a Synergy H1 Monochromator-Based Multi-Mode Microplate Reader (BioTek, Winooski, VT, USA) with excitation and emission wavelength at 485 and 530 nm, respectively. Determination of fluorescence from the DHS expression plasmid was performed as described previously [37]. The background fluorescence from the uninfected erythrocytes was subtracted to yield fluorescence measurements for analysis. The background-corrected SYBR Green I signals from drug-treated parasites were normalized to the average background corrected signal from appropriate control parasites without drug treatment.

The normalized growth data from dose–response assays were analyzed using the drc package [38] in R version 4.0.2 (R Development Core Team, Boston, MA, USA). The two-parameter log-logistic regression model with top and bottom shared among the −GlcN and +GlcN conditions was chosen for comparison of EC_50_ values. The ratio of 50% growth inhibition constants (EC_50_) between −GlcN and +GlcN conditions was determined using the EDcomp in drc. EC_50_ ratios and confidence intervals were plotted using ggplot2 in R.

To determine the effect of GlcN on growth inhibition, parasites were co-treated with 2.5 mM GlcN. The growth inhibitors employed in this experiment were: 6-bromo-*N*-(1*H*-indol-4yl)-1-benzothiophene-2-carboxamide and pyrimethamine as a control (Figure S1, Supplementary Materials). Compounds were diluted in culture medium, in which the maximum concentration of DMSO did not exceed 0.1%. Control wells with no growth inhibitory compound contained 0.1% DMSO. All experiments were performed in quadruplicate (*n* = 4). The maximum dose of the inhibitor compound was 50 µM.

### 4.3. Enzymatic Synthesis of Deoxyhypusine

The first step of EIF5A modification was performed with recombinant, purified EIF5A from *P. vivax* and recombinant, truncated *P. falciparum* DHS [39]. Both plasmodial DHS and *P. vivax* EIF5A were N-terminally histidine-tagged fusion proteins in recombinant *pET-15b* vectors and successfully expressed in *E. coli* BL21(DE3). After cell lysis, purification by nickel-chelate affinity chromatography under native conditions was performed. A subsequent buffer change with 0.1 M glycine/NaOH buffer was employed. The enzyme activity assay was performed in a total volume of 1 mL, containing 25 μg plasmodial DHS, 40 μg purified *P. vivax* EIF5A precursor protein, 4 mM spermidine, 3 mM NAD, 147 μL protease inhibitor cocktail (Roche, Mannheim, Germany) and 0.1 M glycine NaOH buffer and different allosteric inhibitor concentrations ranging from 0.4 µg to 0.8 µg/µL. In parallel, negative controls were performed without the allosteric inhibitor. Incubation took place at 37 °C in a shaker overnight and was stopped by freezing at −80 °C. For further analysis, two-step size exclusion chromatography with Amicon-Ultracel filters (Amicon, Schwalbach, Germany) of 30 kDa and 100 kDa pore size [40] was per-formed to remove the human DHS and concentrate the modified eIF5A (dhp) for further use. Before proceeding to GC/MS analysis, the samples were hydrolyzed for 24 h at 110 °C in 6 NHCl.

### 4.4. GC MS Analysis

The GC/MS method for the analysis of deoxyhypusine was developed according to an approach which was previously described [41]. A total of 200 μL of the peptide hydrolysates was transferred into a glass vial. Thereafter, 125 pmol norvaline was added to the sample as an internal standard. A speedvac vacuum concentrator (Savant ISS110, Thermo Electron Corporation, Karlsruhe, Germany) was employed to remove the solvent. A mixture of 250 µL containing deuterated ethanol and acetyl chloride (85/15 (*v*/*v*)) was used for esterification of the carboxyl group and the sample was derivatized at 110 °C for 20 min. Within the next step, the derivatization reagent was removed at 110 °C by a gentle steam of nitrogen. The amino function was subsequently derivatized using 250 μL of a mixture of trifluoroacetic acid anhydride and trifluoroacetic acid ethyl ester (1:2 (*v*/*v*)). The mixture was added to the sample and heated at 130 °C for 10 min. Removal of the reagent was performed at room temperature by a gentle steam of nitrogen. Next, an additional derivatization step was necessary for the hydroxyl group of hypusine. This was achieved by adding 100 μL hexamethyldisilazane and subsequent heating of the sample for 15 min at 70 °C. A supplementation of 50 μL of dichloromethane followed before the solution was transferred to the GC vial before injection. For GC/MS analysis, an Agilent Technologies 7890 A GC-System with MS detector (MS Agilent Technologies 5975 C inert MSD with Triple-Axis) and a DB5-MS capillary column (30 m × 250 μm i.d. × 0.1 μm film thickness) from Agilent (Waldbronn, Germany) was employed. An injection volume of 1 μL was used and applied in the splitless mode using helium as carrier gas with a flow rate of 1 mL/min. The following temperature program was used: Maintenance of a temperature of 50 °C for 0.75 min. Ramping to 75 °C was achieved with 50 °C/min and kept for 1 min. Next, an increase in the temperature with 6 °C/min to 280 °C was performed with a final hold for 10 min. Selected ion monitoring (SIM) was applied to obtain the chromatogram, i.e., for norvaline *m*/*z* = 126, 168 and 199, and for deoxyhypusine *m*/*z* = 126, 180 and 533, respectively. To quantify the respective substances, the sum of the signal intensities of each of the three ions was accumulated. Determination of the absolute inhibition of *P. falciparum* DHS by the small-molecule inhibitor compound was established, using deoxyhypusine [42] and norvaline (Sigma Aldrich, Munich, Germany) as an internal standard. This method enables quantification of deoxyhypusine in the pmol/μL concentration range. An expression of inhibition in % is possible by both methods described herein.

## 5. Conclusions

6-bromo-*N*-(1*H*-indol-4yl)-1-benzothiophene-2-carboxamide, a potent allosteric inhibitor of human DHS, was found to show weak growth inhibition activity against *P. falciparum*, which cannot be attributed to the targeting of *P. falciparum* DHS. Enzyme inhibition assays confirmed the lack of activity for this compound against *P. falciparum* DHS, pointing to important differences in allosteric binding sites between human and parasite DHS.

## Figures and Tables

**Table 1:** Determination of IC_50_ values using a SYBR Green 1(SG)-based in vitro method.

Strain	Compound	Molecular Weight	IC_50_ (µM)
**Dd2**	6-bromo-*N*-(1*H*-indol-4yl)-1-benzothiophene-2-carboxamide	371.30	46.1
**Dd2**	Amodiaquine	464.80	0.031
**Dd2**	Chloroquine	515.90	0.66
**3D7**	6-bromo-*N*-(1*H*-indol-4yl)-1-benzothiophene-2-carboxamide	371.30	51.5
**3D7**	Amodiaquine	464.80	0.066
**3D7**	Chloroquine	515.90	0.03

**Table 2:** IC_50_ determination for 6-bromo-*N*-(1*H*-indol-4yl)-1-benzothiophene-2-carboxamide in *P. falciparum* and human.

Organism	Protein	IC_50_
*P. falciparum*	DHS	0.340 µM
Human	DHS	0.062 µM [15]

## Data Availability

Not applicable.

## Supplementary Materials

The following supporting information can be downloaded at: https://www.mdpi.com/article/10.3390/molecules27082463/s1, Figure S1: Control dose response growth inhibition curve with pyrimethamine against PfDHS_glmS parasites with co-treatment of 2.5 mM GlcN (blue triangles) or without co-treatment (red circles). Each datapoint represents four independent experiments.
