# Supplementary material for: Investigation of an Allosteric Deoxyhypusine Synthase Inhibitor in P. falciparum
Source: Molecules. 2022 Apr 11;27(8):2463. doi: 10.3390/molecules27082463 (PMC9030622; doi:10.3390/molecules27082463)
Supplement: Supplementary file 1 [file molecules-27-02463-s001.zip › molecules-1576280-supplementary.pdf]

Supplementary File:

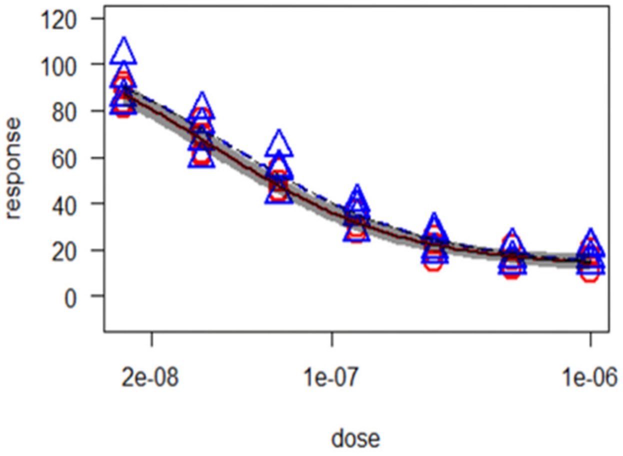

Figure S1. Control dose response growth inhibition curve with pyrimethamine against *Pf*DHS_*glmS* parasites with co-treatment of 2.5 mM GlcN (blue triangles) or without co-treatment (red circles). Each datapoint represents four independent experiments.
